# Infectious and mechanical complications in planned-start vs. urgent-start peritoneal dialysis: a cohort study

**DOI:** 10.1590/2175-8239-JBN-2021-0287en

**Published:** 2022-07-04

**Authors:** João Victor Costa Müller, Daniela Ponce

**Affiliations:** 1 Universidade Estadual Paulista Júlio de Mesquita Filho, Faculdade de Medicina, Botucatu, SP, Brasil

**Keywords:** Peritoneal Dialysis, Renal Replacement Therapy, Peritonitis., Diálise Peritoneal, Terapia de Substituição Renal, Peritonite.

## Abstract

**Background:**

Few studies have compared the infectious and mechanical complications seen in planned-start and urgent-start peritoneal dialysis (PD) patients.

**Objectives:**

To compare the incidence and etiology of mechanical and infectious complications in patients offered planned- and urgent-start PD and assess potential differences in patient survival and time on PD.

**Methods:**

This retrospective cohort study included patients with chronic kidney disease on planned- and urgent-start PD seen from 2014 to 2020 and compared them for mechanical and infectious complications, clinical outcome, death rates, and need to switch to hemodialysis.

**Results:**

Ninety-nine patients on planned-start PD and 206 on urgent-start PD were included. Incidence of exit-site infection (18.9 vs. 17.17%, p=0.71) and peritonitis (24.27 vs. 27.27%, p=0.57) were similar between patients, while pathogens causing peritonitis were different, although non-fermenting Gram-negative bacilli were more commonly seen in the planned-start PD group. Leakage as a mechanical complication and hospitalization were more common among patients needing urgent-start PD (10.68 vs. 2.02%, p=0.0085 and 35.44 vs. 17.17%, p=0.0011, respectively). Patient survival was similar between groups. Cox regression found an association between death and age (HR=1.051, 95% CI 1.026-1.07, p=0.0001) and albumin (HR=0.66, 95% CI 0.501-0.893, p=0.0064), and between peritonitis and a diagnosis of diabetes (HR=2.016, 95% CI 1.25-3.25, p=0.004).

**Conclusion:**

Patient survival and time on PD were similar between the planned- and urgent-start PD groups, while leakage was more frequently seen in the urgent-start PD group. Death was associated with lower albumin levels and older age, while peritonitis was associated with diabetes.

## Resumo

**Antecedentes:**

Poucos estudos compararam as complicações infecciosas e mecânicas relacionadas à diálise peritoneal (DP) de início planejado e não planejado.

**Objetivos:**

Comparar a incidência e etiologia das complicações mecânicas e infecciosas associadas à DP tanto de início planejado quanto não planejado e avaliar comparativamente a sobrevida da técnica e dos pacientes.

**Métodos:**

Estudo de coorte retrospectivo que avaliou pacientes com doença renal crônica em DP não planejada e planejada de 2014 a 2020 quanto às complicações mecânicas e infecciosas e desfechos clínicos óbito ou mudança para hemodiálise.

**Resultados:**

Foram avaliados 99 pacientes em DP planejada e 206 em DP não planejada. Foram semelhantes quanto à incidência de Infecção do Orifício de Saída (18,9x17,17%, p=0,71), peritonite (24,27x27,27%, p=0,57) e diferentes quanto aos agentes etiológicos das peritonites, sendo os bacilos Gram-negativos não fermentadores mais frequentes no grupo planejado. Diferiram quanto à complicação mecânica extravasamento e internação, ambas mais frequentes no grupo não planejado (10,68 x 2,02%, p=0,0085 e 35,44 x 17,17%, p=0,0011, respectivamente). Semelhantes quanto à sobrevida dos pacientes e da técnica. À regressão de Cox, associou ao óbito a idade (HR=1,051, IC 95% 1,026-1,07, p=0,0001) e a albumina (HR=0,66, IC 95% 0,501-0,893, p=0,0064), e à peritonite a presença de diabetes (HR=2,016, IC 95% 1,25-3,25, p=0,004).

**Conclusão:**

A sobrevida da técnica e dos pacientes foi semelhante nos grupos DP planejada e não planejada, enquanto o extravasamento foi mais frequente no grupo de início não planejado. Associaram-se ao óbito menores valores de albumina e maior idade, e à peritonite, o diabetes.

## Introduction

Chronic kidney disease (CKD) is a global public health issue and an alarming epidemic, as it affects 8-16% of the population^1^. The National Kidney Foundation (NKF) divides CKD into five stages. Patients with stage-5 or end-stage renal disease (ESRD) have a GFR <15mL/min/1.73m² and require dialysis^1^.

Patients with ESRD may be offered peritoneal dialysis (PD) or hemodialysis (HD)^2^. Many studies have compared the clinical outcomes of patients prescribed PD vs. HD, with none achieving clear superiority in terms of death. However, patients on HD outnumber patients on PD^3,4^.

PD has been recently considered an option for patients with ESRD in urgent need of starting renal replacement therapy (RRT), particularly in countries with insufficient HD beds/seats^5,6^.

Peritonitis is the most significant complication of PD^7^. According to the literature, the most frequent pathogens involved are *Staphylococcus epidermidis* and *Staphylococcus aureus* ^8^.

Mechanical complications include catheter tip migration, obstructions, and dialysate leakage^9^.

The few studies that have compared patients on planned- vs. urgent-start PD for infectious and mechanical complications often included limited numbers of patients, described controversial results, considered only early complications, and did not explore the etiology of the agents or factors associated with the described outcomes^10-12^. An Australian study described a significant difference in early dialysate leakage and catheter tip migration, with both complications occurring more frequently in the urgent-start PD group. No difference was found in time on PD between groups^10^. Another study performed in Singapore did not find differences in mechanical complications (14 *vs.* 15%, p=1) or patient survival after 180 days of treatment (88 *vs.* 94%, p=0.59)¹¹. In 2019, Wojtaszek et al. reported more frequent mechanical complications in patients on urgent-start PD (29 *vs*. 4%, p*=*0.00005), with leakage described as the most frequent event^13^.

Given the scarcity of HD seats/beds in Brazil, the fact that PD has become an option for patients in need of RRT, and the small number of studies comparing the mechanical and infectious complications seen in individuals on planned- and urgent-start PD, this study aimed to compare the incidence of mechanical and infectious complications associated with planned- and urgent-start PD; identify and compare the factors associated with mechanical and infectious complications; and describe causing factors, patient survival and time on PD relative to each group.

## Methods

### Patients

This retrospective cohort study included patients on planned- and urgent-start PD seen between July 1, 2014 and July 1, 2020 at a dialysis center. Data was extracted from patient electronic charts. The definition of urgent-start PD used in this study is the one proposed by Blake^14^, which comprises patients with previously unknown advanced CKD or with CKD rapidly progressing into ESRD, and patients started on PD within 72 hours of catheter implantation without a history of RRT.

The study enrolled patients with stage-5 CKD on PD. The subjects were divided into two groups:

- Group 1: Planned-start PD - patients followed by a nephrologist prior to RRT prescription, started on PD at least seven days after the implantation of a peritoneal catheter, with trained family members and adapted homes;
- Group 2: Urgent-start PD - patients followed or not by a nephrologist prior to RRT prescription, started on PD within less than 72 hours of catheter implantation, without trained family members or adapted homes, without a history of HD.

After analysis for contraindications and prescription of PD, patients were implanted a Tenckhoff catheter using the Seldinger percutaneous technique as follows: (1) patient lying on their back with a nasal cannula for oxygen supplementation and a saturometer; (2) prophylaxis with intravenous cefazolin 1 g; (3) skin is cleaned with chlorhexidine and drapes are placed; (4) catheter is measured from the pubic symphysis to the first cuff two centimeters to the left of the umbilical scar; (5) an incision is made to the skin and the deep places are dissected; (6) a Tenckhoff catheter is implanted using the Seldinger technique (peritoneal puncture performed with a 16G needle followed by dilation of the muscle and peritoneal planes); (7) a PD fluid infusion and drainage test is performed; (8) a metallic guidewire is advanced; (9) a tunnel is made through subcutaneous cell tissue, keeping the cuff about 3 cm away from the exit site; (10) connectors are placed; (11) skin is sutured using Nylon 3.0 wire; (12) bandages are placed^14,15^.

The patients were followed by the same medical team from the moment the peritoneal catheter was implanted and PD was started until they either died, were sent for transplantation, recovered kidney function, or switched to HD due to treatment failure or mechanical/infectious complications.

This study complied with the principles set out in Resolution 196/96 for research with human beings and was approved by the local Ethics Committee for Research with Human Beings (certificate CAEE 30691120.2.0000.5411). The patients were provided information about the research protocol, content and relevance of the study and joined only after signing an informed consent term.

The mechanical complications considered were catheter migration or obstruction leading to difficulty draining the dialysate (<80% of the infused volume); dialysate leaking through the surgical incision or the catheter exit site; drainage difficulties or bleeding requiring a blood transfusion; pain; need to surgically relocate the catheter; and hospitalization.

The infectious complications analyzed were exit-site infection (ESI) defined as purulent effusion draining out of the catheter exit site^16^; and peritonitis, defined as presence of abdominal pain and cloudy effluent, confirmed through effluent total and differential cell counts (>100 polymorphonuclear cells, with 50% neutrophils) or a positive culture^16^.

### Statistical Analysis

Data were entered in a spreadsheet, checked for typos, and analyzed on SAS for Windows (version 9.2: SAS Institute, Cary, NC, USA, 2012).

Considering an alpha error of 5%, a beta error of 20%, a statistical power of 80%, and a detection of a 20% difference in mortality between the groups, the calculated sample size was 59 patients per group.

A descriptive analysis was first performed for all included patients, followed by the calculation of measures of central tendency and scatter for continuous variables and of frequencies for categorical variables. The chi-squared test was used to establish comparisons between group categorical variables, while continuous variables were compared via the t-test when they followed a normal distribution or with the Mann-Whitney test when not. Survival curves were produced for time on PD and patients, considering the time it took for patients to switch to HD or die, respectively, in addition to a curve for survival free of mechanical complications and peritonitis, which show how long it took for patients to develop mechanical complications or peritonitis, respectively, using the Kaplan-Meier estimator. A Cox regression was used to identify factors associated with mechanical and infectious complications. Statistical significance was assigned to differences with a p <0.05.

## Results

A total of 306 were analyzed, of which 206 (67.5%) were on urgent-start PD and 99 on planned-start PD. Table 1 shows a comparison of group characteristics. Diabetes was the most common underlying disease. Groups were similar in terms of death rate (17.48 vs. 14.14%, p=0.4614) and treatment failure (27.67 vs. 28.28%, p=0.9110), as shown in Table 1. The urgent-start PD group was significantly younger (56.17±16.61 vs. 60.27±16.55, p=0.03); C-reactive protein (CRP) and phosphate levels on admission were higher in the urgent-start PD group, while hemoglobin levels on admission were higher in the planned-start PD group, as seen in Table 2.

**Table 1:** Clinical characteristics of patients on urgent- and planned-start peritoneal dialysis

	Urgent-start PD (n = 206)	Planned-start PD (n = 99)	p
AGE^*^	56.17±16.61	60.27±16.55	**0.03**
Males (%)	109(52.91)	52(52.52)	0.9494
PTH^**^	242(128-401)	195.59(123-322.75)	0.11
CRP^**^	1.2(0.5-2.2)	0.6(0.5-1.45)	**0.0026**
hb^**^	9.35(8.5-10.5)	11.2(10.3-12.4)	**<0.0001**
Creatinine clearance^**^	7.02(4.8-9.73)	7.26(4.68-10.52)	0.07
Albumin^**^	3.4(3-3.7)	3.7(3.2-4)	**<0.0001**
Phosphate^**^	6.05(5-7.6)	5.15(4.4-6.2)	**<0.0001**
UNDERLYING DISEASE (%)			
Diabetes	64 (31.06)	26(26.53)	0.3889
Hypertension	39 (18.93)	27(27.55)	0.0977
2 OR MORE COMORBIDITIES (%)	94 (45.63)	40(40.82)	0.3891
INDICATION (%)			
Uremia	150 (72.82)	71(71.72)	0.8407
Hypervolemia	9 (4.37)	7(7.07)	0.3217
DEATH (%)	36(17.48)	14(14.14)	0.4614
TREATMENT FAILURE (%)	57(27.67)	28(28.28)	0.911

ESI: exit-site infection; PTH: parathyroid hormone; CRP: C-reactive protein; HB: hemoglobin;

* Mean±standard deviation,

** Median (quartiles)

**Table 2:** Types of complications seen in patients on urgent- and planned-start peritoneal dialysis

	Urgent-start PD N = 206	Planned-start PD N = 99	p
Mechanical complications (%)	56 (27.18)	20 (20.2)	
Drainage difficulty	24(11.65)	13(13.13)	0.71
Leakage	22(10.68)	2(2.02)	**0.008**
Catheter tip migration	9(4.37)	4(4.04)	0.68
Bleeding	1(0.48)	0(0)	0.54
Need of catheter surgical relocation	33 (16.02)	19 (19.19)	0.49
Hospitalization	73(35.44)	17(17.17)	**0.001**
Infectious complications (%)	89 (43.20)	44 (44.44)	
ESI	39 (18.93)	17 (17.17)	0.71
Peritonitis	50 (24.27)	27 (27.27)	0.57

ESI: exit-site infection

In regard to mechanical complications, the groups had similar proportions of cases of catheter tip migration and bleeding (p>0.05), but were significantly different in leakage cases, with subjects in the urgent-start PD group being more affected (10.68 vs. 2.02%, p=0.0085). Hospitalization was also more frequent in the urgent-start PD group (35.44 vs. 17.17%, p=0.001), as shown in Table 2.

The analysis of etiological factors tied to ESI and peritonitis revealed that the groups were similar to each other. Gram-positive pathogens prevailed in cases of ESI, while Gram-negative ones were more prevalent in cases of peritonitis in both groups, as seen in Table 3. Non-fermenting Gram-negative bacilli were more common in the planned-start PD group (0% vs. 11.11%, p=0.01), as shown in Table 3.

**Table 3:** Pathogens causing exit-site infection and peritonitis in patients on urgent- and planned-start peritoneal dialysis

	Urgent-start PD (n = 39)	Planned-start PD (n = 17)	p
ESI PATHOGENS			
CoNS - (%)	14(35.9)	6(35.29)	0.96
*S. aureus* (%)	6(15.38)	4(23.53)	0.46
Negative culture (%)	5(12.82)	1(5.88)	0.44
Enterobacteria (%)	6(15.38)	3(17.65)	0.83
NFGNB (%)	4(10.26)	0(0)	0.17
PERITONITIS PATHOGENS	Urgent-start PD (n = 50)	Planned-start PD (n = 27)	p
CoNS - (%)	5(10)	7(25.93)	0.06
*S. aureus* (%)	8(16)	3(11.11)	0.55
Negative culture (%)	15(30)	6(22.22)	0.46
Enterobacteria (%)	15(30)	5(18.52)	0.27
NFGNB (%)	0(0)	3(11.11)	**0.01**
*C. Albicans* (%)	5(10)	1(3.70)	0.32

ESI: exit-site infection; CoNS: coagulase-negative staphylococci; NFGNB: non-fermenting Gram-negative bacilli

Univariate analysis found associations between death and age (55.76±16.53 vs. 67.48 ± 13.8, p<0.0001), bacteremia (3.53 vs. 20%, p<0.0001), and hospitalization (25.1 vs. 52%, p=0.0001), as shown in Table 4.

**Table 4:** Clinical characteristics of patients on peritoneal dialysis based on progression (death and treatment failure)

	Death			Treatment failure		
	No (n = 255)	Yes (n = 50)	p	No (n = 220)	Yes (n = 85)	p
AGE^*^	55.76±16.53	67.48±13.8	**<0.0001**	58.07±16.97	56.73±15.89	0.35
Males (%)	131(51.37)	29(58)	0.3909	118(53.64)	42(49.41)	0.50
PTH^**^	213(96.9-381.4)	124.5(28.37-221.87)	**0.0007**	185.25(80.37-347.25)	194(93-337)	0.20
CRP^**^	0.5(0-1.4)	1.1(0.5-3.77)	**0.0008**	0.5(0-1.32)	0.8(0.5-1.9)	**<0.0001**
HB^**^	10(8.9-11.35)	9.65(8.4-10.97)	0.1218	9.95(8.9-11.2)	9.7(8.6-11.2)	0.32
Creatinine clearance^**^	6.96(4.42-10.06)	6.48(4.4-10.09)	0.4098	7(4.77-9.9)	6.44(3.6-10.24)	0.07
Albumin^**^	3.5(3-3.9)	3.2(2.63-3.8)	**0.0002**	3.5(3-3.9)	3.3(2.8-3.8)	**0.01**
Phosphate^**^	5.6(4.6-7)	5.85(4.87-7.52)	0.1457	5.6(4.6-6.92)	5.7(4.7-7.5)	0.22
Urgent-start PD (%)	170(66.67)	36(72)	0.4614	149(67.73)	57(67.06)	0.91
UNDERLYING DISEASE (%)						
Diabetes	72(28.24)	19(38)	0.1676	58(26.36)	33(38.82)	**0.03**
Hypertension	58(22.75)	8(16)	0.2896	46(20.91)	20(23.53)	0.61
2 OR MORE COMORBIDITIES (%)	110(43.14)	25(50)	0.3717	99(45)	36(42.35)	0.67
INDICATION (%)						
Uremia	186(72.94)	35(70)	0.6703	155(70.45)	66(77.65)	0.20
Hypervolemia	15(5.88)	1(2)	0.2602	7(3.18)	9(10.59)	**0.0093**
Type of mechanical complication (%)						
Drainage difficulty	30(11.76)	7(14)	0.658	19(8.64)	18(21.28)	**0.002**
Leakage	19(7.45)	5(10)	0.5405	15(6.82)	9(10.59)	0.27
MECHANICAL COMPLICATION (%)	65(25.49)	12(76)	0.8245	47(21.36)	30(35.29)	**0.0092**
RELOCATION SURGERY (%)	45(17.65)	7(14)	0.5306	30(13.64)	22(25.88)	**0.0108**
ESI (%)	45(17.65)	12(24)	0.292	45(20.45)	18(21.18)	0.88
ESI PATHOGEN (%)						
CoNS	14(31.11)	7(58.33)	0.0824	12(26.67)	10(55.56)	**0.02**
*S. aureus*	8(17.78)	2(16.67)	0.9284	12(26.67)	2(11.11)	0.17
Negative culture	4(8.89)	2(16.67)	0.4354	6(13.33)	1(5.56)	0.37
Enterobacteria	8(17.78)	1(8.33)	0.4253	6(13.33)	3(16.67)	0.73
NFGNB	4(8.89)	0(0)	0.2841	3(6.67)	1(5.56)	0.87
PERITONITIS (%)	61(23.92)	16(32)	0.2292	43(19.55)	35(41.18)	**0.0001**
BACTEREMIA (%)	9(3.53)	10(20)	**<0.0001**	14(6.36)	5(5.88)	0.87
PERITONITIS PATHOGEN (%)						
CoNS	9(14.75)	3(18.75)	0.6949	8(18.6)	5(14.29)	0.61
*S. aureus*	9(14.75)	1(6.25)	0.3678	5(11.63)	5(14.29)	0.72
Negative culture	16(26.23)	6(37.5)	0.3744	10(23.26)	11(31.43)	0.41
Enterobacteria	16(26.23)	4(25)	0.9205	13(30.23)	7(20)	0.30
NFGNB	2(3.28)	1(6.25)	0.5846	3(6.98)	0(0)	0.11
*C. Albicans*	5(8.20)	1(6.25)	0.796	1(2.33)	5(14.29)	**0.0487**
NEW PERITONITIS EPISODES (%)	18(7.06)	5(10)	0.4714	11(5)	12(14.12)	**0.0069**
HOSPITALIZATION (%)	64(25.1)	26(52)	**0.0001**	62(28.18)	28(32.94)	0.41
REASON TO SWITCH TO HD (%)						
Infection	40(15.69)	0(0)	**0.0027**	0(0)	39(45.88)	**<0.0001**
Mechanical complication	4(1.57)	0(0)	0.3727	0(0)	41(48.24)	**<0.0001**

ESI: exit-site infection; CoNS: coagulase-negative staphylococci; NFGNB: non-fermenting Gram-negative bacilli; PTH: parathyroid hormone; CRP: C-reactive protein; HB: hemoglobin;

* Mean±standard deviation,

** Median (quartiles)

The following factors were associated with PD failure: mechanical complication (21.36 vs. 35.29%, p=0.009); peritonitis (19.55 vs. 41.18%, p=0,0001); and C. Albicans infection (2.33% vs. 14.29%, p=0.048), as shown in Table 4.

Multivariate analysis using Cox regression found that death was associated with older age (HR=1.0519, 95% CI 1.0264-1.078, p=0.0001); higher albumin levels at the start of therapy were a protective factor (HR=0.6692, 95% CI 0.5013-0.8934, p=0.0064); and diabetes was associated with the development of peritonitis (HR=2.016 95% CI 1.2505-3.2504, p=0.004), as shown in Table 5.

**Table 5:** Cox regression for outcomes of peritonitis, mechanical complication, treatment failure, and death

	HR	95% CI	p
PERITONITIS			
Urgent-start PD	1.1504	0.6884 a 1.9224	0.5928
Age	1.0057	0.9907 a 1.0210	0.4584
Albumin	1.0439	0.7638 a 1.4268	0.7873
HB	0.9528	0.8440 a 1.0757	0.4349
PCR	0.9975	0.9847 a 1.0106	0.7092
Diabetes mellitus	**2.0160**	**1.2505 a 3.2504**	**0.0040**
MECHANICAL COMPLICATION			
Urgent-start PD	0.6536	0.3767 a 1.1340	0.1304
Age	0.9942	0.9806 a 1.0081	0.4126
Albumin	0.9023	0.6925 a 1.1757	0.4464
HB	1.0294	0.9155 a 1.1574	0.6284
PCR	0.9982	0.9846 a 1.0120	0.7985
Diabetes mellitus	0.9205	0.5426 a 1.5616	0.7587
TREATMENT FAILURE			
Urgent-start PD	0.8473	0.5313 a 1.3515	0.4867
Diabetes mellitus	1.2746	0.8142 a 1.9954	0.2887
CRP	1.0039	0.9986 a 1.0092	0.1492
Albumin	0.8393	0.6778 a 1.0393	0.1082
Hypervolemia	2.0164	0.9939 a 4.0906	0.0520
DEATH			
Urgent-start PD	0.5762	0.2922 a 1.1365	0.1117
Age	1.0519	1.0264 a 1.0780	**0.0001**
Albumin	0.6692	0.5013 a 0.8934	**0.0064**
HB	1.0099	0.8867 a 1.1504	0.8815
CRP	1.0027	0.9896 a 1.0160	0.6860
Diabetes mellitus	1.7611	0.9596 a 3.2318	0.0677

HB: hemoglobin; CRP: C-reactive protein

**Figure 1:**
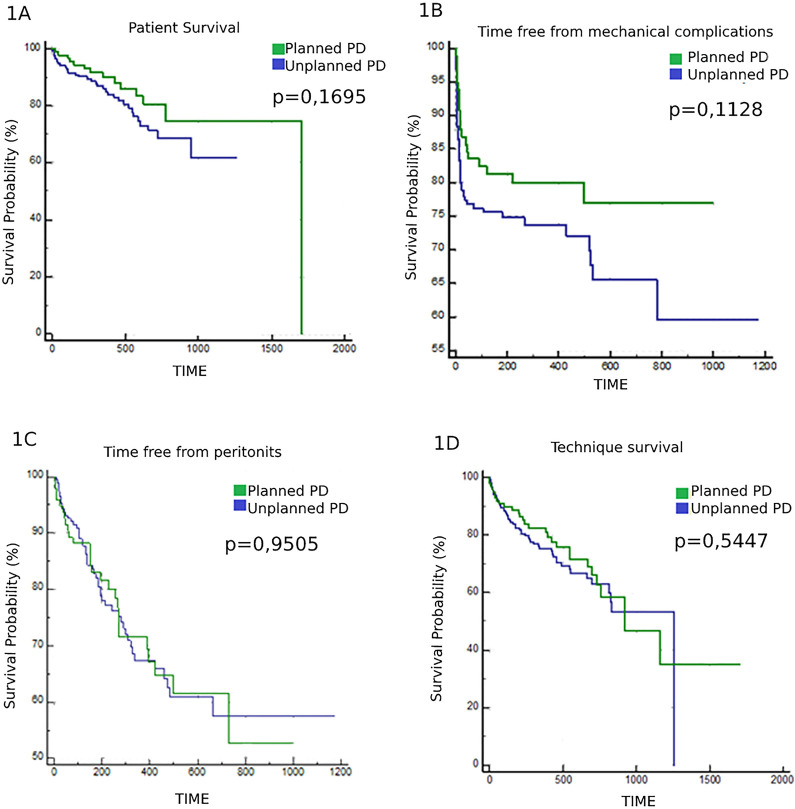
Patient survival (1A), survival free of mechanical complications (1B) survival free of peritonitis (1C), time on peritoneal dialysis (1D), urgent- and planned-start patients.

Patient survival (p=0.1695), time on PD (p=0.5447), survival free of mechanical complication (p=0.1128) and survival free of peritonitis (p=0.9505) were similar between groups, as seen in Figure 1.

## Discussion

In the period covered by our study, the vast majority of the patients were started on urgent PD, reflecting the service’s strategy to deal with the scarcity of HD seats/beds since 2014.

The definition of urgent-start PD was revised by Blake^14^ and described as a strategy used for patients with previously unknown advanced CKD or with CKD rapidly progressing into ESRD, and patients started on PD within 72 hours of catheter implantation without a history of RRT. In our study, urgent-start PD meant patients followed or not by a nephrologist prior to RRT prescription, started on PD within less than 72 hours of catheter implantation, without trained family members or adapted homes and without a history of HD, i.e., subjects residing within the parameters defined by Blake.

The groups differed in CRP and phosphate levels at the start of dialysis, with higher levels seen in the urgent-start PD group, while higher levels of hemoglobin and albumin were observed in the planned-start PD group. The difference in albumin, hemoglobin, and phosphate levels in the urgent-start PD group may be explained by the fact that patients in this group had not been followed by a nephrologist before the start of PD and, thus, had not been provided with nutrition advice, iron supplementation, chelating agents, or erythropoietin.

The groups were similar in terms of incidence of mechanical and infectious complications, but differed in etiology of infectious agents and leakage, the latter a more frequent event in the urgent-start PD group.

Controversial results have been described in the literature concerning the comparison of mechanical and infectious complications in patients undergoing planned- and urgent-start PD. For two years, Lobbedez et al.^17^ followed 60 patients on urgent-start dialysis, 34 on PD and 26 on HD. Only two of the patients on urgent-start PD had mechanical or infectious complications and no difference was seen regarding mechanical or infectious complications in patients started immediately on dialysis vs. patients given a rest after having the peritoneal catheter implanted. In a prospective observational study, Alkatheeri et al.^18^ analyzed 30 patients on urgent-start PD for mechanical and infectious complications. Ten percent of all patients had leakages in the first week of treatment, but in none was therapy discontinuation required. Another 20% of the patients suffered with catheter tip migration, which was repaired without catheter replacement or need to switch patients to a different treatment mode. No cases of peritonitis or ESI were observed after catheter insertion. In 2019, Wojtaszek et al. compared the short and long term outcomes of 35 patients on urgent-start PD and 94 on planned-start PD and found that leakage was the most frequent complication in the urgent-start group^13^.

A study initiated in Singapore in July 2015 included 17 patients on urgent-start PD and 33 patients on planned-start DP. The authors did not find differences between the groups in terms of hospitalization, mechanical complications, or patient survival after 180 days, although the urgent-start PD group had higher creatinine and urea levels at the start of therapy^11^. In 2018, Nayak et al. studied 24 patients on planned-start and 32 on urgent-start dialysis and reported similar results for leakage, catheter obstruction, and time on dialysis within 90 days^12^.

An Australian study compared patients on urgent- and planned-start PD for early complications and survival. In a group of 104 patients (26 on urgent- and 78 on planned-start PD), the authors found significant differences in leakage and catheter tip migration, with greater incidence in the urgent-start group. They found no difference in infectious complications. Time on PD was not different either, although the urgent-start group had a greater rate of mechanical complications. The data gathered in our study were similar to the findings described in the Australian publication, in that leakage as a mechanical complication was more prevalent in the urgent-start PD group, while no difference was found in infectious complications, patient survival, or time on PD between groups.

An earlier study reported an incidence of peritonitis of 42%, with 72.6% of the cases occurring within the first six months of treatment^7^. In our study, the overall incidence of peritonitis was 25.25%. We found no difference in rates of peritonitis, treatment failure, patient survival, time on PD, or number of peritonitis-free days using the Kaplan-Meier estimator.

According to the literature, the most common etiological agents are skin pathogens, such as *Staphylococcus epidermidis* and *Staphylococcus aureus*, with contamination occurring during catheter changes. However, studies performed at our service have shown a prevalence of Gram-negative pathogens^8^, a finding corroborated in the present study. The only difference resided in the greater prevalence of infection involving non-fermenting Gram-negative bacilli (NFGNB) in the planned-start PD group. No difference was seen in the pathogens found in ESI. This may be explained by the use of a prophylactic cream (gentamycin) on the catheter exit site^19^.

We also looked into the factors associated with unfavorable clinical outcomes in the two groups of patients. Urgent-start PD was not a predictor of unfavorable outcome. Cox regression analysis found that diabetes mellitus was associated with peritonitis, while age and albumin level on admission were predictors of death.

Age as a factor related to death has been associated with greater comorbidity and shorter survival in patients on dialysis^20^. Hypoalbuminemia reflects both malnutrition and a state of chronic inflammation; it has also been associated with cardiovascular events and shorter survival in individuals on dialysis^21^. Specifically in PD, hypoalbuminemia may explain the greater prevalence of leakage and infection, caused either by a weaker abdominal wall due to malnutrition or impaired healing due to inflammation.

A number of factors weigh in on the interpretation of these findings. Following patients prior to the start of dialysis is a key element to improving their nutritional status and finding the best time to start therapy, thereby preventing malnutrition from setting in and albumin levels from decreasing, which decreases the incidence of mechanical and infectious complications and improves treatment success rates and patient survival.

Finally, our study showed that time on PD, patient survival, and incidence of mechanical and infectious complications were similar in both groups. The urgent-start PD group had lower rates of hospitalization and complications by leakage, while incidence of infection by non-fermenting Gram-negative bacilli was higher in the planned-start PD group.

Our results are supported by the findings of a number of prior studies that suggested that PD is a viable and safe alternative for patients on urgent-start dialysis and a valuable tool to increase the number of patients treated with chronic PD. PD is a treatment option that should be offered to every patient without contraindications who urgently need to start dialysis.
